# MCF-Net: Personnel and machinery detection model for complex downhole drilling environments

**DOI:** 10.1371/journal.pone.0320653

**Published:** 2025-05-29

**Authors:** Zhupeng Jin, Hongcai Li

**Affiliations:** School of Mining Engineering, Heilongjiang University of Science and Technology, Harbin, China; Universiti Teknologi Petronas: Universiti Teknologi PETRONAS, MALAYSIA

## Abstract

Detection of personnel and machinery in the drilling environment of underground coal mines is crucial to the safe production of coal. However, the existing detection models seriously affect the accuracy of the detection models due to the problems of insufficient light and mutual occlusion in the underground. To address these problems, this study proposes a lightweight downhole personnel and machinery detection model (MCF-Net), which aims to solve the above problems while improving the detection accuracy and speed of the model. In this study, YOLOv10 is used as the baseline model, and the PSA module in the backbone network is replaced by designing a lightweight attention mechanism MLBAM, which improves the model’s multi-scale feature extraction capability, enhances the model’s detection performance of mutually occluded objects and reduces the model’s complexity. In the neck network, C2f is reconstructed to get C2f-DualConv based on DualConv, and the group convolution technique is used to extract downhole image features, which can effectively overcome the influence of interference factors such as insufficient illumination in the downhole. Finally, Focaler-CIoU is introduced to reconstruct the IoU loss function, which can accurately locate the downhole objects. In addition, this study conducts experiments on a real downhole borehole dataset, and the results show that compared with the baseline model, MCF-Net improves the accuracy by 0.013 for mAP @ 0.5 and 0.046 for mAP @ 0.5:0.95, reduces the model complexity by 2.27MB for Params and 8.4 for GLOPs, and improves the inference speed by FPS is improved by 11.83 f/s.

## 1. Introduction

With the rapid development of industry and economy, coal has become an important part of global energy consumption [1]. However, certain hazards inevitably exist in the coal mining environment, such as gas explosions, roofing, and internal and external fires [2,3], which pose a serious threat to the safety of miners. Therefore, in order to promote the safety of coal production, it is necessary to introduce advanced detection technology to detect the coal mining process.

In recent years, with the rapid development of deep learning research, some researchers have used target detection techniques in deep learning to detect important targets in underground environments. For example, Cao et al. performed miners’ unsafe behavior identification by using skeleton spatiotemporal maps constructed from multi-frame human key points [4], which can effectively avoid coal mining accidents triggered by miners’ unsafe behaviors. Kong et al. detected coal mine fire smoke by improving YOLOv8s [5], which can detect mine fires in time to manage them.

Although some progress has been made in the detection research of underground coal mines, there is a lack of certain research for specific underground drilling scenarios. Underground drilling in coal mines is a key measure to solve hidden disasters such as gas and water damage [6,7]. When drilling, real-time detection of the surrounding personnel and machinery can effectively ensure the safety of drilling work and reveal the hidden disaster-causing factors in the coal mine production process. Downhole drilling scenarios have limited computing resources and high real-time requirements, making it difficult to deploy complex, large-scale models underground [8]. At the same time, the scenario often suffers from insufficient lighting and mutual occlusion of personnel and machinery, which seriously affects the accuracy of the model.

YOLOv10 achieves low computational overhead and low inference latency of the model by designing an overall efficiency-accuracy-driven model strategy and training without non-maximum suppression (NMS) for consistent dual-tasking, which meets the requirements of downhole drilling scenarios with limited computational resources and real-time performance. However, for the poor image quality and limited grey scale range caused by insufficient downhole lighting, the model is difficult to extract sufficient detail information from the image. Although YOLOv10 is adaptable to low-quality images, it is prone to misdetection and omission under such extreme environment as downhole. At the same time, downhole drilling scenarios have mutual occlusion between personnel and mechanical equipment [9]. When different objects are in overlapping or close contact, YOLOv10 may lose object detection or misclassify multiple objects.

Recently, many deep learning based low light enhancement algorithms have been proposed by researchers to address the image degradation problem caused by shooting under low light conditions.Hu. et al. improve the quality of low-light images by processing multi-channel information of low-light images without the need of colour change based on CNN to better learn the non-linear mapping between low light and normal light [10]. Liu.et al. faced with the difficulty of obtaining high quality pairwise data dependence in low-light enhancement tasks, proposed LEA- by designing entropy inspired kernel selective convolutional sub-network (EIKS) and illumination-concentration-transfer (IAT) module [11]. Net, which explores the relationship between image characteristics and image quality from the data itself to achieve low-quality image enhancement.

Introducing the attention mechanism is the main solution to the object occlusion problem, Li et al. introduced the attention mechanism to CNN’s proposed ACNN algorithm and evaluated it on real-world occluded face datasets, the results show that ACNN improves the recognition accuracy of both non-occluded and occluded faces [12]. Jin et al proposed a framework called Attribute Based Shifted Attention Network (ASAN) re-ID framework for occluded person detection, by evaluating it on seven datasets of different kinds, it is obtained that the shifted-attention-based occluded person detection has better accuracy and generalization for occlusion problem [13].

Inspired by the above solutions, this paper proposes an efficient and low-complexity target detection model, MCF-Net, by improving YOLOv10.Firstly, by fusing the CBAM and MLCA attention mechanisms, a new lightweight attention mechanism, MLBAM, is proposed to replace the PSA module in the backbone network, which enhances the model’s ability of extracting the information of the image channels and effectively improves the the detection performance of the model when facing the downhole occlusion problem. Secondly, C2f is reconstructed into C2f-DualConv, which uses group convolution technique to process the feature maps simultaneously, which can suppress the influence of downhole dimming and improve the accuracy of the model. Finally, Focaler-CIoU is introduced to reduce the effect of too much training on background classes and improve the accuracy of edge regression, focusing on difficult-to-detect targets. This enables MCF-Net to accurately locate and detect targets in complex downhole drilling environments.

## 2. Related work

Target detection is a fundamental task of computer vision, which has been widely used in various real-world scenarios [14], such as autonomous driving, robot vision, video surveillance, etc. Currently, target detection can be categorized into two types of detectors: ‘two-stage detectors’ and ‘one-stage detectors’.

“two-stage detectors” are CNN-based detectors with a ‘coarse-to-fine’ detection framework. RCNN [15] is an early two-stage detector, which firstly selects a set of object candidate frames re-scales the contents of the selected frames to a fixed-size image, and finally recognizes the object categories through SVM by comparing with the trained CNN model to extract features. CNN model to extract features, and finally recognize the object categories by SVM.SPPN [16] solves the problem of redundant feature computation in RCNN by computing the feature mapping once for the whole image and then generating a fixed length of arbitrary regions to train the detector. Faster-RCNN [17] can train the detector faster by fusing RCNN and SPPN. However two-stage detectors can lead to low detection speed of the model due to the detection framework being divided into two stages.

“one-stage-detectors” means that detection is achieved through a single-stage framework. YOLO [18] was proposed in 2015 by Joseph et al. It is the first single-stage detector in the era of deep learning. Compared to the two-stage detector YOLO follows a different paradigm, which provides a high improvement in detection speed but decreases in recognition accuracy, particularly for the small target problem. The subsequent successor versions of YOLO YOLOv3 [19] YOLOv4 [20], YOLOv5 [21], YOLOv7 [22], and YOLOv8 [23] are focusing on solving the problem in YOLOv10 by eliminating the non-maximum suppression (NMS) and optimizing the various model components, which enables YOLOv10 to significantly reduce the computational overhead while improving the accuracy of detection [24].

In recent years, with the continuous development of deep learning, the target detection technology has been continuously applied to coal production scenarios to ensure the safe production of coal. For example, Mao et al. improved YOLOv5s by introducing Ghost convolution and Soft-NMS anchor frame screening method, which reduced the number of parameters of YOLOv5s and solved the problem of leakage due to overlapping of gangplanks, and realised the intelligent identification of the interference state between the drum and the gangplank of underground coal miners [25]. Wang et al. proposed YOLO-BS for the problem of YOLOv4 unbalanced sample of large lumps of coal on underground scraper conveyor, which can effectively detect the large lumps of coal on underground scraper conveyor and solve the problem of coal congestion in underground transport process [26]. Fu et al. proposed an improved coal gangue detection algorithm for YOLOv8 for the problems of poor real-time performance and high computational cost of YOLOv8, which can accurately classify coal and gangue [27].

## 3. Materials and methods

**Fig 1:**
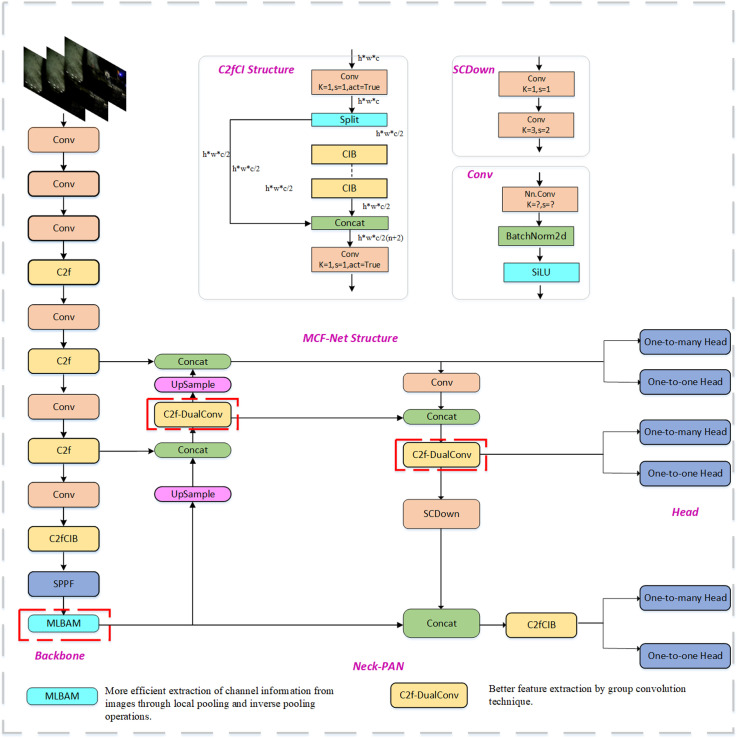
MCF-Net structure.

In this study, a lightweight downhole personnel and machinery detection model (MCF-Net) is proposed to ensure the safety of downhole drilling work by taking the downhole drilling environment as the research background, which is characterized by insufficient light and serious mutual occlusion problems. The network structure is shown in Fig 1. The input image size of the model is kept at 640×640×3 and consists of three parts: backbone, neck, and detect.

In order to solve the problem of low recognition accuracy caused by insufficient light in the complex downhole drilling environment, this study combines the Convolutional Block Attention Module (CBAM) and Mixed Local Channel Attention (MLCA) to propose a new lightweight attention mechanism (Mixed Local Block Attention Module (MLBAM), which replaces the PSA module in the YOLOv10m backbone network, enhances the spatial channel extraction capability through local pooling and inverse pooling operations, improves the detection accuracy of the model in the face of the under-illuminated environment, and reduces the complexity of the model. At the same time, C2f-DualConv is used to replace the baseline model YOLOv10m feature fusion module C2f, and the group convolution mechanism of DualConv 3×3 convolution kernel and 1×1 convolution kernel is used to efficiently arrange the convolution filters in the parallel layout of the input feature mapping dimensions, which can efficiently take care of the spatial feature extraction ability of the large-size convolution kernel and the computational efficiency of the small-size convolution kernel to ensure that the model is lightweight and at the same time solves the problem of spatial feature extraction. The model is lightweight and at the same time solves the problem of low model detection accuracy due to mutual occlusion in the downhole. Finally, the Focaler-CIoU function is introduced to reconstruct the loss function, so that the model can focus more on different regression samples and accurately locate the detection target in the detection task under the drilling environment.

### 3.1 MLBAM attention mechanisms

The PSA attention mechanism in the YOLOv10m network architecture can effectively extract important features across dimensions in multi-scale information and channel attention vectors. However, in the complex environment of downhole drilling, the ability to extract spatial information is relatively insufficient. In this study, a novel lightweight attention mechanism MLBAM is proposed based on CBAM combined with MLCA, which can effectively extract geospatial information features in downhole drilling environments by enhancing channel information.

#### 3.1.1 CBAM attention mechanisms.

**Fig 2:**
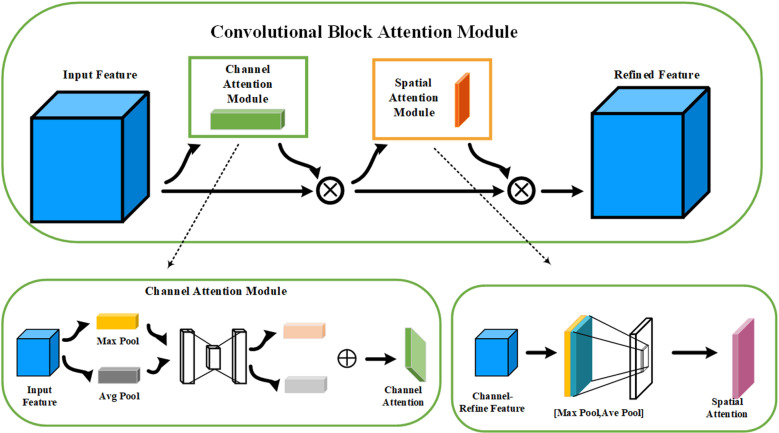
CBAM structure.

The CBAM attention mechanism [28] consists of two sub-modules, CAM (Channel Attention Module) and SAM (Spatial Attention Module). The CAM module extracts the channel information of the image and the SAM extracts the spatial information of the image, and the network structure of the two integrations enables the CBAM to capture multi-scale image The network structure is shown in Fig 2. The network structure is shown in Fig 2.

#### 3.1.2 MLCA attention mechanisms.

**Fig 3:**
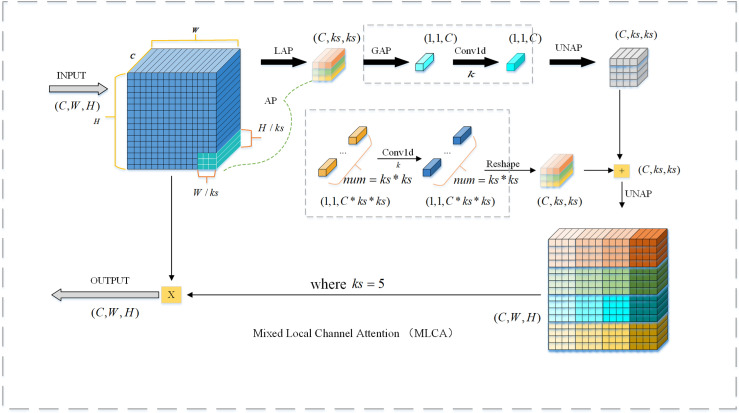
MLCA structure.

The input features of the MLCA attention mechanism [29] extract the local information of the image input features through local pooling and inverse pooling operations and capture the inter-channel spatial information through the relationship between the one-dimensional convolutional attention channel and the k-nearest neighbor channels. Finally, MLCA achieves comprehensive extraction of image features by fusing local and global information. The structure and working principle of the MLCA attention mechanism is shown in Fig 3.

**Fig 4:**
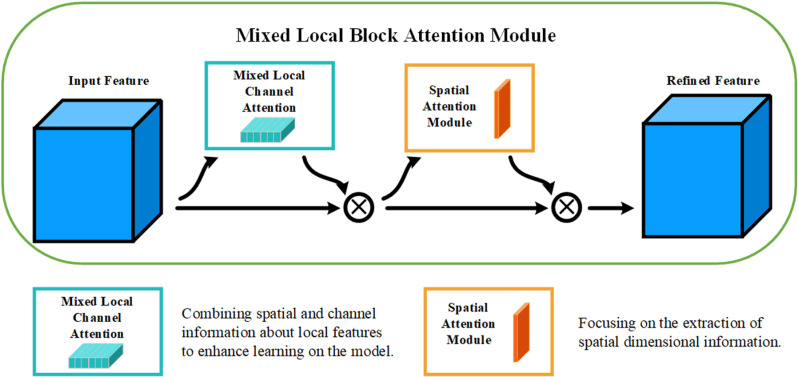
MLBAM structure.

The attention mechanism of MLBAM adopts the overall network structure of CBAM, which enhances the important areas in the feature map through spatial attention and channel attention. However, in the complex environment of downhole drilling, there are some limitations in the ability of traditional CBAM to extract spatial information, and CBAM can pay attention to the important spatial locations effectively, but it has insufficient ability to process multi-scale information, and it can not be adequately adapted to the complex environment. geospatial information in complex environments. In this study, MLCA is introduced into CBAM instead of the channel attention module, so that MLBAM can extract channel information in images more efficiently through local pooling and anti-pooling operations in the complex environment of coal mine drilling. The MLBAM network architecture is shown in Fig 4.

### 3.2 C2f-DualConv

**Fig 5:**
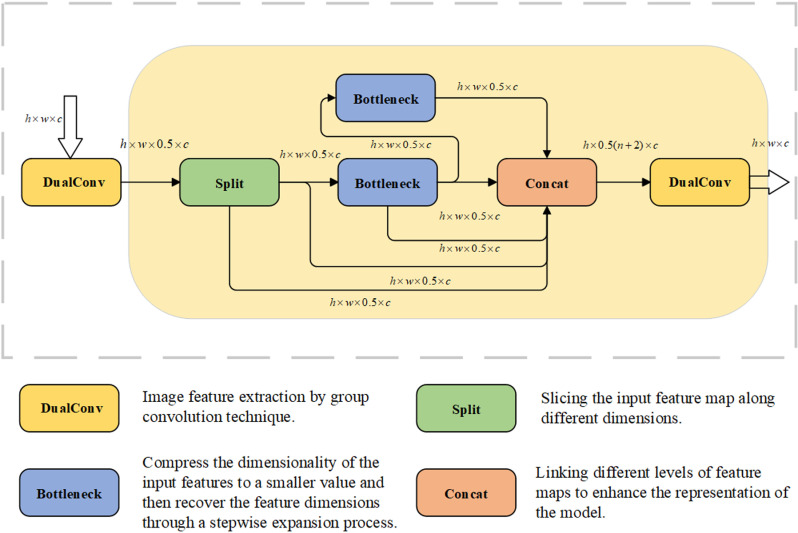
C2f-DualConv structure.

**Fig 6:**
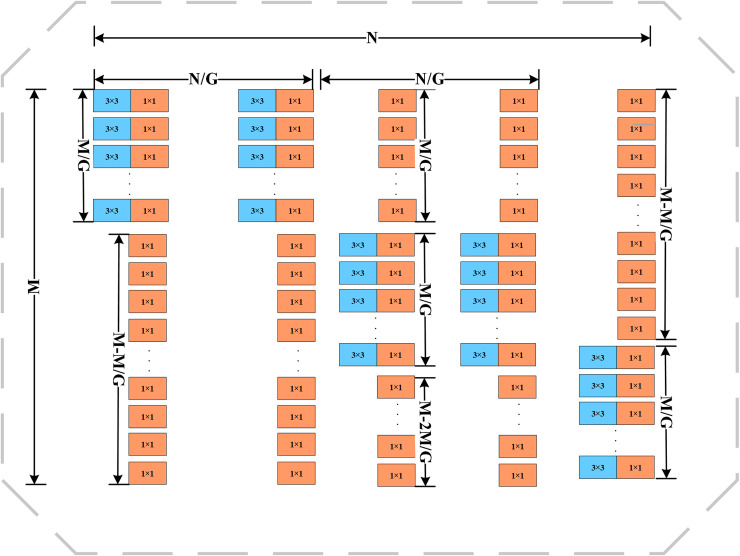
DualConv structure.

In order to achieve a lighter weight and more accurate model in the coal mine underground drilling environment, DualConv [30] is used in this paper to reconstruct the traditional Conv convolution module to improve C2f, and it is named C2f-DualConv. The C2f-DualConv network structure is shown in Fig 5. Compared with the traditional C2f, C2f-DualConv processes the same input feature map channel by using both 1×1 and 3×3 dual convolution kernels through the DualConv operation, which enables the model to better capture spatial features at different scales. In addition, C2f-DualConv employs the group convolution technique to efficiently arrange the convolution filters, thus reducing the number of parameters of the model and improving the computational efficiency.The DualConv network structure is shown in Fig 6.

### 3.3 Focaler-CIoU

Loss functions are used to measure the degree of overlap between the prediction frames generated by the detection model and the manually labelled real frames to optimise the parameters of the model and improve the detection accuracy. In target detection tasks, common loss functions such as IoU (Intersection over Union) loss are usually used to measure the overlap between the predicted and real frames. However, the traditional IoU loss function cannot effectively guide the model to focus on the difficult-to-detect regions when dealing with dim and highly occluded objects downhole, resulting in a degradation of model performance.

In this paper, Focaler-CIoU Loss [31] is introduced, which is able to enhance the model’s focus on hard-to-detect regions through the focal loss mechanism, and compared with the traditional IoU loss function, Focaler-CIoU isable to dynamically adjust the focus of loss to focus more attention on hard-to-detect targets, thus improving the model’s performance in complex scenes. Focaler-CIoU Loss is defined as shown in Equation 1.


$$L_{Focaler-CIoU}=L_{CIoU}+IoU-IoU^{Focaler}$$ (1)

The IOUFocaler uses a linear interval mapping approach to reconstruct the IoU loss as shown in Equation 2.


$$IoU^{\text{F}ocaler}= \{\begin{array}{l} 0,Iou<d\\ \frac{\left(IoU-d\right)}{u-d}\\ 1,IoU>u \end{array}),d\leq IoU\leq u$$ (2)

## 4. Experiments and results

### 4.1 Introduction to the experiment

In this section, the dataset, experimental environment, and model training parameters used for the experiments are described and finally, the evaluation metrics used to assess the experiments are presented.

#### 4.1.1 Dataset.

**Fig 7:**
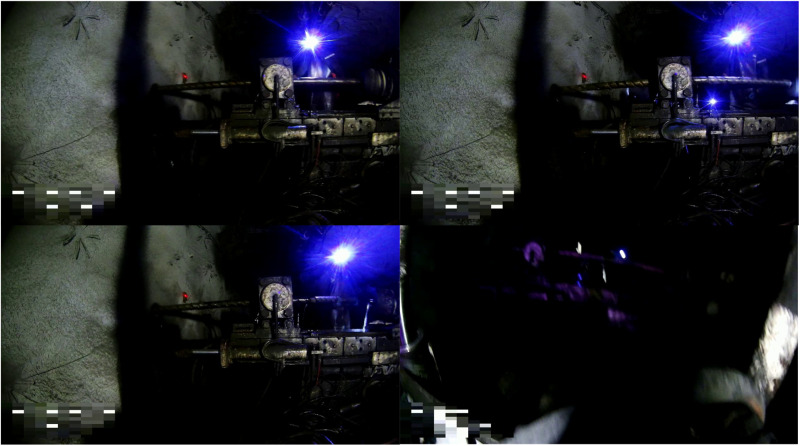
Representative dataset images.

The ZK_Dataset [32] dataset used in this study was collected from a mine in Binzhou City, Shaanxi Province, with a large collection time span, involving multiple working face drilling scenarios, covering changes in light intensity, shooting angle, target occlusion, and other dimensions, and the drilling videos collected were randomly intercepted by 30~50 frames through the Opencv library, and in order to adapt to the complex working environment of underground drilling in coal mines, occluded In order to adapt to the complex working environment of coal mine drilling, the obscured images are selected to construct ZK_Dataset, and the total number of images is 28886. All the labels in the dataset are labelled by LabelImg in YOLO format, with five categories: gripper, drill pipe, coal miner, mining helmet and chuck. In order to ensure the high quality of the dataset, several experts in the field of coal mining participated in the sampling of the dataset. The representative data images are shown in Fig 7.

#### 4.1.2 Experimental environment and training strategies.

The MCF-Net model training and testing experimental platform proposed in this study is shown in Table 1.

**Table 1:** Experimental platform-related configuration.

Parameters	Configuration
CPU	Intel(R) Xeon(R) Gold 6152 CPU * 10核
GPU	NVIDIA GeForce RTX 3090
GPU Memory	24G
CUDA	12.1
Deep learning framework:	Python 3.10.14 + Pytorch2.0.1

In this experiment, the use of hyperparameters for training, testing and validation of all models is kept consistent to ensure comparable experimental results. In order to select the optimal hyperparameter combination, systematic tuning was performed in this study, and cross-validation and experimental comparisons were made to ensure that each hyperparameter configuration could achieve the best performance under different training conditions. In this study, the initial learning rate is set to 1 × 10–2, the random seed is taken as 1, and the MultiStepLR strategy is used to dynamically adjust the learning rate. In this experiment, the learning rate will decay at the 100th epoch and 200th epoch moments with a decay coefficient of 0.1. With the MultiStepLR strategy, the learning rate gradually decays during the training process, which helps the model to be more finely tuned when it is close to convergence, and at the same time avoids training instability due to too high a learning rate. For the optimiser configuration, a stochastic gradient descent (SGD) optimiser with a momentum value of 0.937 is used. The momentum strategy helps to reduce the fluctuations during gradient updating, speeds up the convergence rate, and improves the training stability. Finally, an early stopping strategy [33] was used, i.e., training was stopped when the performance of the three Epoch models did not improve during the training process. The key hyperparameters of the training process are shown in Table 2.

**Table 2:** The setting of key hyperparameters for model training.

Parameters	Value
Epoch	300
Initial learning rate	1×10^-2^
Final learning rate	1×10^-4^
Momentum	0.937
Batch Size	32
Input image size	640×640
Optimizer	SGD
Patience	3

The dataset for mechanical detection of personnel in downhole drilling work environment is divided into the training set, validation set, and test set, 7:2:1 format, of which 20,220 are in the training set, 5,777 are in the validation set, and 2,889 are in the test set.

#### 4.1.3 Evaluation indicators.

In this study, detection precision and model complexity are used to evaluate the performance of MCF-Net. In terms of detection accuracy, precision, and recall, mAP@0.5, and mAP@0.5:0.95 are introduced [34]. Where precision refers to the probability of actual positive samples among those predicted to be positive, which is calculated as shown in Equation 3.


$$Precision=\frac{TP}{TP+FP}$$ (3)

Where TP (True Positive) represents the number of sample correct model predictions that are correct and FP (False Positive) represents the number of sample incorrect model predictions that are correct.

Recall refers to the actual correct samples predicted as correct samples, which is calculated as shown in Equation 4, where FN (False Positive) is worth the number of samples correctly predicted incorrectly.


$$Recall=\frac{TP}{TP+FN}$$ (4)

AP (Average Precision) is equal to the area under the precision-recall curve and is calculated as shown in Equation 5:


$$AP=\int_{0}^{n}\left(Precision\right)d\left(Recall\right)$$ (5)

The mAP ((Mean Average Precision) is the weighted average of APs across all categories and is used to measure the detection performance of the model across all categories. The calculation formula is shown in Equation 6.


$$mAP=\frac{\sum_{0}^{j}AP}{j}$$ (6)

where j denotes the jth detection category in the target detection task. In addition, mAP@ 0.5 denotes the recognition accuracy when the IoU threshold is 0.5. In the target detection task, IoU is used to measure the overlap between the predicted bounding box and the true bounding box,mAP@ 0.5:0.95 refers to the accuracy of the model detection when the IoU threshold is from 0.5 to 0.95 [35].

In terms of model complexity, GLOPs, Params, and FPS evaluation metrics are used to evaluate the model, GLOPs refer to the amount of computation (computational time complexity), which can be used to measure the complexity of the algorithm [36]. Params is the size of the model parameter count [37], and FPS refers to the number of frames of the image that can be processed by the hardware in one second, by which the metrics can be used to evaluate the model in terms of the inference speed under the same hardware [38].

### 4.2 Experimental results

In this study, the ZK_Datasets dataset is used to evaluate the detection effect of the MCF-Net model in a complex downhole drilling environment. To ensure that the test results of different models in the experiment are not affected by factors other than the model, both the experimental environment and the training strategy of the model are kept consistent during the training process.

#### 4.2.1 Comparison experiment.

In this section, we compare the MCF-Net proposed in this study with four mainstream target detection models, yolov5m, yolov7, yolov8m, and yolov10m, to evaluate the performance of the MCF-Net detection model. The comparison results are shown in Table 3. As can be seen from the results in the table, MCF-Net has the highest accuracy in the comparison results with the accuracy metrics mAP @0.5 and mAP @0.5:0.95 of 0.961 and 0.684, respectively. Compared with yolov7 mAP@ 0.5, which ranks second in terms of accuracy, mAP@ 0.004 and mAP@ 0.5:0.95 are improved by 0.037. In terms of model complexity metrics, MCF-Net has 14.18M and 55.0 in terms of Params and GLOPs, which is a lower demand on hardware resources compared to other models, with Params@0.5 and mAP@0.5:0.95 compared to second-ranked yolov10m, which is 0.961 and 0.684 in terms of accuracy. yolov10m with a decrease of 2.27MB in Params and 8.4 in GLOPs. In terms of inference speed, MCF-Net shows a faster speed with an FPS of 260.34 f/s.

**Table 3:** Comparison of experimental results.

Model	mAP@0.5	mAP @0.5:0.95	Params(M)	GLOPs	FPS
yolov5m	0.95	0.638	25.04	64	282.29
ylov7	0.957	0.647	36.50	103.2	285.72
yolov8m	0.952	0.645	25.84	78.7	250.11
ylov10m	0.948	0.638	16.45	63.4	248.51
**MCF-Net**	**0.961**	**0.684**	**14.18**	**55.0**	**260.34**

In downhole drilling scenarios, factors such as limited computational resources and environmental changes affect the deployment and accuracy of the model. MCF-Net’s lightweight design and low hardware requirements enable it to run stably on low-power and high-efficiency hardware, which solves the problem of high demand for hardware resources in traditional target detection models. In addition, MCF-Net effectively copes with complex environmental factors such as insufficient light and occlusion downhole through multi-scale feature extraction and group convolution techniques, thus ensuring its robustness and accuracy in the special environment of downhole drilling.

The above experimental results show that compared with other models, MCF-Net is a detection model that balances complexity and accuracy, and is able to cope with challenges such as hardware limitations and environmental changes in the underground coal mine environment.

In order to further verify the performance improvement of MCF-Net compared to the baseline model, this study conducted a t-test on the results of the two models. Five fixed random seeds (1, 7, 42, 76, 95) were set up during the experiments and the experiments were conducted under each seed separately. The experimental results show that the results of baseline model on mAP@0.5 are 0.948, 0.945, 0.952, 0.953, and 0.947, while the results of MCF-Net under the same metrics are 0.961, 0.963, 0.960, 0.961, and 0.959, respectively.The T-test was calculated to obtain a T-value of -7.41, a P-value less than 0.001, rejecting the original hypothesis (i.e., there is no significant difference between the two models) and concluding that MCF-Net is significantly better than the baseline model in terms of performance.

#### 4.2.2 Ablation experiment.

In order to verify the impact of different improvement strategies on the detection performance of the baseline model, ablation experiments are conducted by gradually adding improvement strategies to the baseline model, and the results of the ablation experiments are shown in Table 4. The experimental results show that the introduction of MLBAM improves mAP @0.5 and mAP @0.5:0.95 by 0.007 and 0.013, respectively, while in terms of model complexity, Params is reduced by 1.25 and GLOPs by 1.0. In terms of inference speed, the model achieves a notable improvement of 2.85 frames per second in inference speed.The introduction of MLBAM is used for the improvement of The introduction of MLBAM is used to improve the PSA feature extraction module in the backbone network of the yolov10m baseline model, which improves the multi-scale feature extraction capability of the model by enhancing the channel information, and solves the problem of personnel occlusion under the complex downhole drilling conditions.

**Table 4:** Ablation results.

Model	mAP@0.5	mAP @0.5:0.95	Params(M)	GLOPs	FPS
yolov10m(baseline)	0.948	0.638	16.45	63.4	248.51
+MLBAM	0.955	0.651	15.20	62.4	251.36
+C2f-DualConv	0.951	0.642	15.43	56.0	257.55
+MLBAM+ C2f-DualConv	0.957	0.677	14.18	55.0	260.02
**+MLBAM+ C2f-DualConv+Focaler-CIoU(Ours)**	**0.961(+)**	**0.684(+)**	**14.18(+)**	**55.0(+)**	**260.34(+)**

Based on the introduction of MLBAM, the neck network is designed C2f-DualConv, which solves the problem of dim and disturbing noise in the mine re-drilling environment by processing the input image feature maps simultaneously with 1×1 and 3×3 dual convolution kernels. Meanwhile, with the introduction of MLBAM and C2f-DualConv, the models mAP @0.5 and mAP @0.5:0.95 are improved by 0.009 and 0.039 compared with the baseline model, the Params are reduced by 2.27M, the GLOPs are reduced by 8.4, and the inference speed FPS is improved by 11.51 f/s. The ZK_Dataset data used in this study focuses on a representative occlusion situation downhole, and MCF-Net introduces Focaler-CIoU to reconstruct the IoU loss, which focuses more on the location information of the low-resolution pictures downhole so that the detection model can accurately localize the manned machinery downhole.

**Fig 8:**
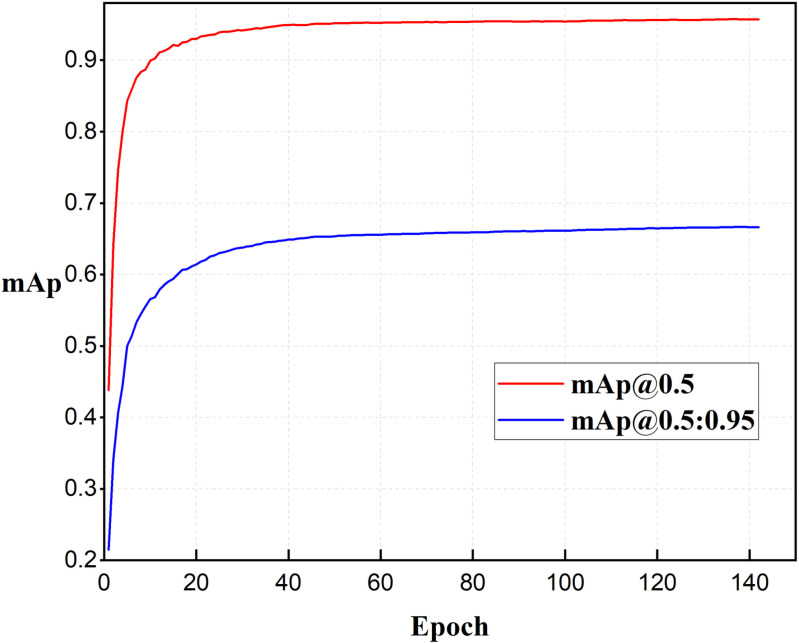
Model training accuracy rise curve.

MCF-Net reduces the GLOPs and Params by 13.25% and 13.8%, respectively, and improves the mAP @0.5 and mAP @0.5:0.95 by 1.37% and 7.21%, respectively. MCF-Net can reduce the difficulty and cost of deploying the model on the mobile side, so that the algorithm can satisfy real-time performance and have higher accuracy, and its training process is shown in Fig 8. The training process is shown in Fig 8.

#### 4.2.3 Visualization of experimental results.

**Fig 9:**
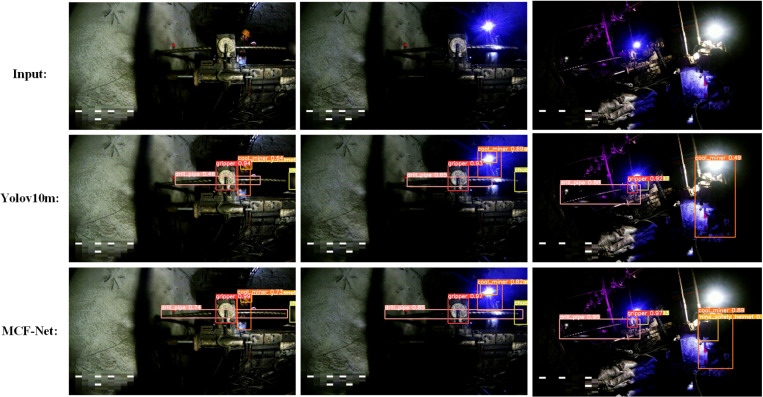
Comparison of different models.

In order to evaluate the performance of the MCF-Net detection model more intuitively, we use a consistent test set to compare yolov10m and MCF-Net, and both models are able to correctly detect people and machinery in the complex drilling environment of the mine, as shown in Fig 9. The improved MCF-Net has a strong multi-scale extraction ability, can face the occlusion problem in the underground, and has a strong accuracy rate for the occluded detection objects. At the same time, MCF-Net can effectively suppress the irrelevant background information in the complex environment of the downhole, so that the model can accurately localize the people and machinery in the downhole. For example, in Fig 9, MCF-Net has improved the detection accuracy of both coal_miner and mine_safety_helmet in the face of drilling machinery occlusion, and at the same time, as can be concluded from the comparison of Fig 9 two rows, three columns, and three rows, three columns pictures, MCF-Net is able to solve to a certain extent the poor detection effect brought about by downhole illumination reasons. Finally, from the comparison of two rows and one column with three rows and one column and two rows and two columns with three rows and three columns in Fig 9, MCF-Net is able to locate more accurately for the drill_pipe detection target.

**Fig 10:**
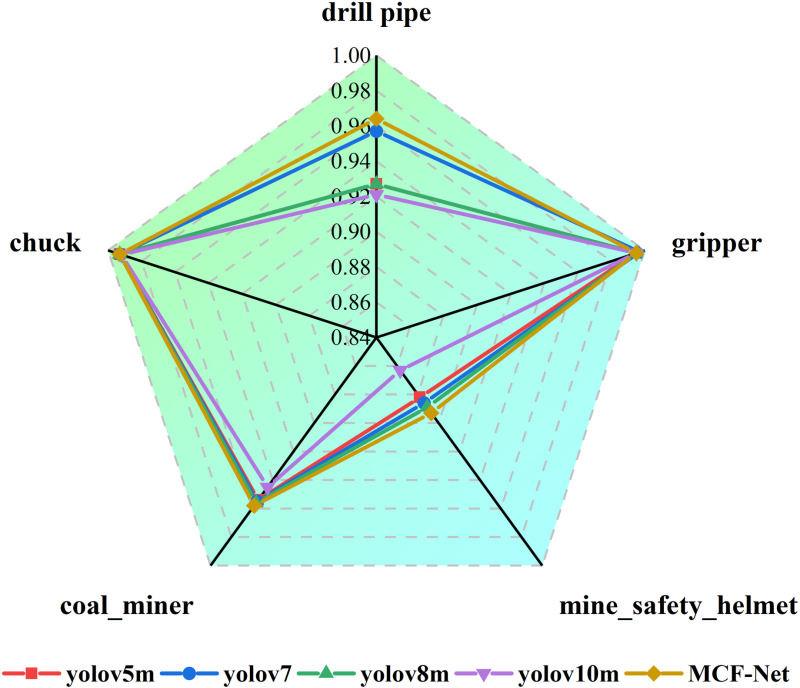
Radar chart of test results by category.

In order to evaluate the detection advantage of MCF-Net over other models for different categories in the drilling environment, yolov5m, yolov7-tiny, yolov8m, and yolov10m are selected to plot the detection effects of different categories, as shown in Fig 10. Compared with other models, MCF-Net shows strong detection ability in all five detection categories, in which the mAP @0.5 for the drill pipe category is 0.964 which is 0.037, 0.007, 0.037, 0.043 higher than that of yolov5m, yolov7-tiny, yolov8m, and yolov10m, and the mAP @0.5 for the grip category is 0.037, 0.007, 0.037, and 0.043, respectively, and the mAP @0.5 of the drill pipe category is 0.964, 0.007, 0.007, 0.037, and 0.043, respectively. mAP @0. 5 of 0.995 remains basically unchanged compared to the other models, mAP @0.5 of 0.893 for the mining safety helmet category improves by 0.011, 0.007, 0.004, 0.03 compared to yolov5m, yolov7-tiny, yolov8m, and yolov10m, respectively, and mAP @0.5 of 0.007, 0.004, 0.03 for the coal miner category improves by 0.037, 0.007, 0.037, 0.043, and 0.043, respectively, for the gripper category. mAP @0.5 is 0.958 compared to yolov5m, yolov7-tiny, yolov8m and yolov10m, which is improved by 0.004, 0.003, 0.002, 0.013, and mAP @0.5 is 0.993 for the chuck category, which is basically stable compared to other models.

**Fig 11:**
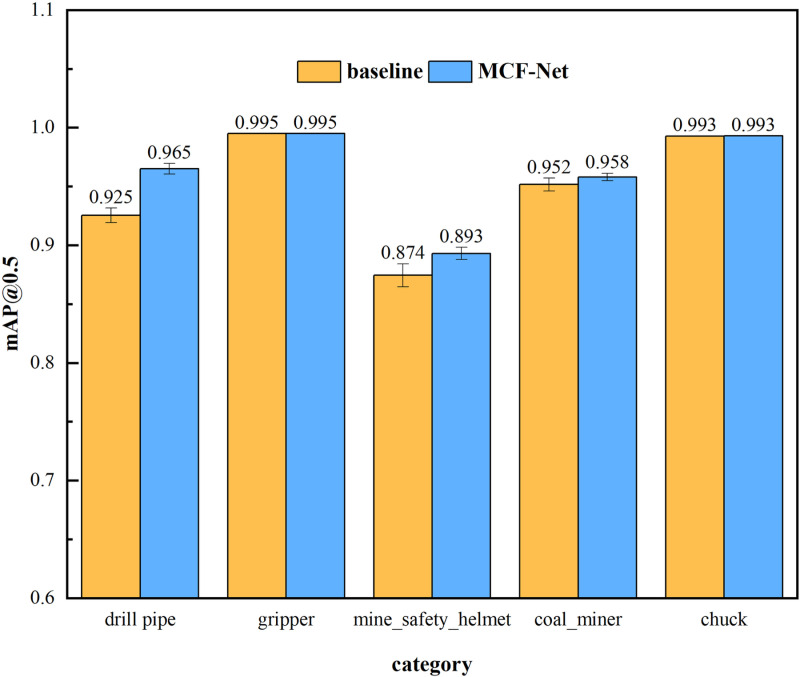
Comparison of detection stability between baseline model and MCF-Net under different categories.

In order to assess the stability and robustness of the model more comprehensively, this study calculated the mean and standard deviation of each type of MCF-Net versus the baseline model yolov10m for multiple random seeds, as shown in Fig 11. In contrast, the standard deviation of MCF-Net is smaller in each category, indicating that it has higher generalisation ability to the complex scenarios of downhole drilling environment.

In addition, in the detection task of the mining helmet category, since helmets in underground environments are usually equipped with torches, the strong light interference may lead to low contrast in the target area, thus affecting the detection accuracy. Therefore, the detection accuracy of this category is relatively low. However, compared to the baseline model YOLOv10m, MCF-Net still achieves better detection performance in this category (an improvement of about 0.03).

## 5. Conclusions

Personnel and machinery detection in underground drilling environments is an important part of coal mine safety production. In view of the problems of insufficient illumination and serious mutual occlusion of people and machinery in an underground drilling environment, this study proposes a lightweight underground personnel and machinery detection model (MCF-Net) for real-time monitoring in an underground drilling environment. By combining CABM and MLCA to design MLBAM to replace the PSA module in the yolov10m backbone network, the model can extract the image features of the complex downhole environment at multiple scales, and better connect the contextual information, so that the model has a better performance in the face of the downhole personnel and machinery occlusion problem. In addition, the C2f-DualConv obtained by reconstructing the C2f in the neck network through the DualConv enables the MCF-Net to extract image features through the convolutional group technique, which effectively solves the problem of dimness and high interference noise in the downhole. Finally, Focaler-CIoU is introduced to reconstruct the IoU loss function, so that the model pays more attention to the position information in the image during the training process, so that the model can more accurately locate the personnel and machinery downhole.

The experimental results show that in terms of model accuracy, mAP @0.5 improves by 0.013 and mAP @0.5:0.95 improves by 0.046 compared to the baseline model. In terms of model complexity, MCF-Net Params and GLOPs decrease by 2.27MB and 8.4, respectively, while the model achieves a notable improvement of 11.83 frames per second in inference speed, having lower parameter counts and operations while increasing the inference speed, which means it can be more simply deployed in hardware.

Overall, the MCF-Net target detection model proposed in this study is a detection model that balances complexity and accuracy. It provides a more applicable method for the detection of personnel machinery in the complex drilling environment under the mine.

Although, MCF-Net has demonstrated superior target detection performance in the complex environment of coal mine underground, there are still limitations in the performance of the model in some specific scenes and conditions. In the coal mine underground, especially in the case of miners wearing helmets, the strong light source from the mining lamps will lead to overexposure or reflection of the image. Although the performance in complex environments is enhanced by multi-level feature fusion, the details of the helmet may be lost in areas with strong light or reflections, resulting in the model failing to accurately identify the target. In addition, the MCF-Net model is mainly targeted at downhole drilling scenarios and may be difficult to directly migrate to other underground environments, such as roadway boring faces. In these environments, despite the presence of similar low-light and complex structures, there may be specific disturbances in each environment that affect the performance of target detection. Therefore, future research will focus on improving the robustness and scalability of models in complex environments and exploring more general model architectures.
